# Identification of specific and common diagnostic antibody markers for gastrointestinal cancers by SEREX screening using testis cDNA phage library

**DOI:** 10.18632/oncotarget.24963

**Published:** 2018-01-01

**Authors:** Sohei Kobayashi, Takaki Hiwasa, Takahiro Arasawa, Akiko Kagaya, Sayaka Ishii, Hideaki Shimada, Masaaki Ito, Masae Suzuki, Masayuki Kano, Bahityar Rahmutulla, Kouichi Kitamura, Yuji Sawabe, Hideo Shin, Masaki Takiguchi, Fumio Nomura, Hisahiro Matsubara, Kazuyuki Matsushita

**Affiliations:** ^1^ Department of Frontier Surgery, Graduate School of Medicine, Chiba University, Chiba 260-8670, Japan; ^2^ Department of Laboratory Medicine & Division of Clinical Genetics and Proteomics, Chiba University Hospital, Chiba 260-8670, Japan; ^3^ Department of Biochemistry and Genetics, Graduate School of Medicine, Chiba University, Chiba 260-8670, Japan; ^4^ Clinical Research Center, Chiba University Hospital, Chiba 260-8670, Japan; ^5^ Department of Gastroenterological Surgery, School of Medicine, Toho University, Tokyo 143-8541, Japan; ^6^ Department of Molecular Oncology, Graduate School of Medicine, Chiba University, Chiba 260-8670, Japan; ^7^ Department of Molecular Diagnosis, Graduate School of Medicine, Chiba University, Chiba 260-8670, Japan; ^8^ Department of Neurosurgery, Higashi Funabashi Hospital, Chiba 274-0065, Japan; ^9^ Divisions of Clinical Mass Spectrometry and Clinical Genetics, Chiba University Hospital, Chiba 260-8670, Japan

**Keywords:** cancer-testis antigen, SEREX, esophageal squamous cell carcinoma, AlphaLISA, gastrointestinal cancers

## Abstract

The present study was planned to identify novel serum antibody markers for digestive organ cancers. We have used screening by phage expression cloning and identified novel fourteen antigens in this experiment. The presence of auto-antibodies against these antigens in serum specimens was confirmed by western blotting. As for auto-antibodies against fourteen antigens, AlphaLISA (amplified luminescence proximity homogeneous assay) assay was performed in the sera of gastrointestinal cancers patients to confirm the results. Serum antibody levels against these fourteen recombinant proteins as antigens between healthy donors (HD) and esophageal squamous cell carcinoma (ESCC) patients, gastric cancer (GC), or colon cancer (CC) were compared. The serum levels of all fourteen auto-antibodies were significantly higher in ESCC and GC than those of HD. Among those auto-antibodies, except ECSA2 and CCNL2, were also detected significantly higher levels in CC than those of HD. Receiver operating curve (ROC) revealed similar results except CCNL2 in CC. AUC values calculated by ROC were higher than 0.7 in auto-antibodies against TPI1, HOOK2, PUF60, PRDX4, HS3ST1, TUBA1B, TACSTD2, AKR1C3, BAMBI, DCAF15 in ESCC, auto-antibodies against TPI1, HOOK2, PUF60, PRDX4, TACSTD2, AKR1C3, BAMBI, DCAF15 in GC, and auto-antibodies against TPI1, HOOK2, PUF60 in CC. AUC of the combination of HOOK2 and anti-p53 antibodies in ESCC was observed to be as high as 0.8228. Higher serum antibody levels against ten antigens could be potential diagnostic tool for ESCC. Higher serum antibody levels against eight antigens could be potential diagnostic tool for GC, and serum antibody levels against three antigens could be potential diagnostic tool for CC.

## INTRODUCTION

There is a specific common mechanism of expressing some molecules (e.g., CEA) (these have been confirmed to be expressed in embryonic stage) during gastrointestinal tumorigenesis. However, there are few diagnostic biomarkers that are able to specifically detect and discriminate various cancers at an early stage. Recent research in the field of cancer therapeutic strategy suggests that there are few biomarker candidates that are able to predict the therapeutic effects. Moreover, these could also function as companion diagnostic tools [1]. The recently developed serum anti-p53 antibodies test was useful for the detection of superficial ESCC [2]. As positive rate of serum anti-p53 antibody was around 30%, other serum antibodies were necessary for a combinational test to improve positive rate and diagnostic accuracy.

The serological identification of antigens by recombinant cDNA expression cloning (SEREX) is an effective screening method for identification of serum antibody-type tumor markers [3]. SEREX could be utilized for the immune-screening of cDNA libraries prepared from tumor specimens with either autologous or allogeneic sera. Furthermore, sequencing the isolated cDNA clones easily identified antigens. This made SEREX suitable for the large-scale screening of tumor antigens. SEREX has been applied to various human tumor types and has identified more than 1000 novel tumor antigens (SEREX antigens) [4]. We have previously performed large-scale SEREX screenings and identified numerous ESCC SEREX antigens. Furthermore, 21 antigens were reported to have included the tumor suppressor p53, the oncoproteins phosphatidylinositol 3-kinase, and stathmin [5–13]. Anti-p53 antibody marker has been utilized for not only ESCC but also gastric cancer (GC) as well as colon cancer (CC). Further, the antibody levels against other SEREX antigens might be responsible for GC and CC. Thus, we examined whether antibody markers against ESCC SEREX antigens were common among digestive organ cancers.

## RESULTS

### Identification of SEREX antigens recognized by sera of patients with ESCC

Expression cloning using the sera of patients with ESCC has identified 14 clones. Some of these clones were identified using λZAP II library. They were triosephosphate isomerase 1 (TPI1) (Accession number: NM_000365), hook microtubule-tethering protein 2 (HOOK2) (Accession number: NM_001100176) [9, 11], peroxiredoxin 4 (PRDX4) (Accession number: NM_006406), heparan sulfate (glucosamine) 3-O-sulfotransferase 1 (HS3ST1) (Accession number: NM_005114), tubulin alpha-1B (TUBA1B) (Accession number: NM_006082), tumor-associated calcium signal transducer 2 (TACSTD) (Accession number: NM_002353) [5, 9], aldo–keto reductase family 1 member C3 (AKR1C3) (Accession number: NM_003739), BMP and activin membrane-bound inhibitor homolog (BAMBI) (Accession number: NM_012342), DDB1- and CUL4-associated factor 15 (DCAF1) (Accession number: NM_138353), phosphodiesterase 4D-interacting protein (PDE4DIP) (Accession number: NM_001198834) [10], esophageal carcinoma SEREX antigen-1 (ECSA1) [11], ECSA2 [11], cyclin L2 (CCNL2) (Accession number: NM_030937) [10, 12], and poly(U) binding splicing factor 60 (PUF60) (Accession number: NM_078480.2) [1, 13, 14]. TPI1, PRDX4, HS3ST1, TUBA1B, AKP1C3, BAMBI, and DCAF1 were identified using testis cDNA library. However, they have not yet been reported. Recombinant proteins were expressed in *E. coli* as GST-fusion proteins and were purified by affinity-chromatography using Glutathione Sepharose.

### Confirmation of the presence of serum antibodies in patients with ESCC by western blotting

**Figure 1:**
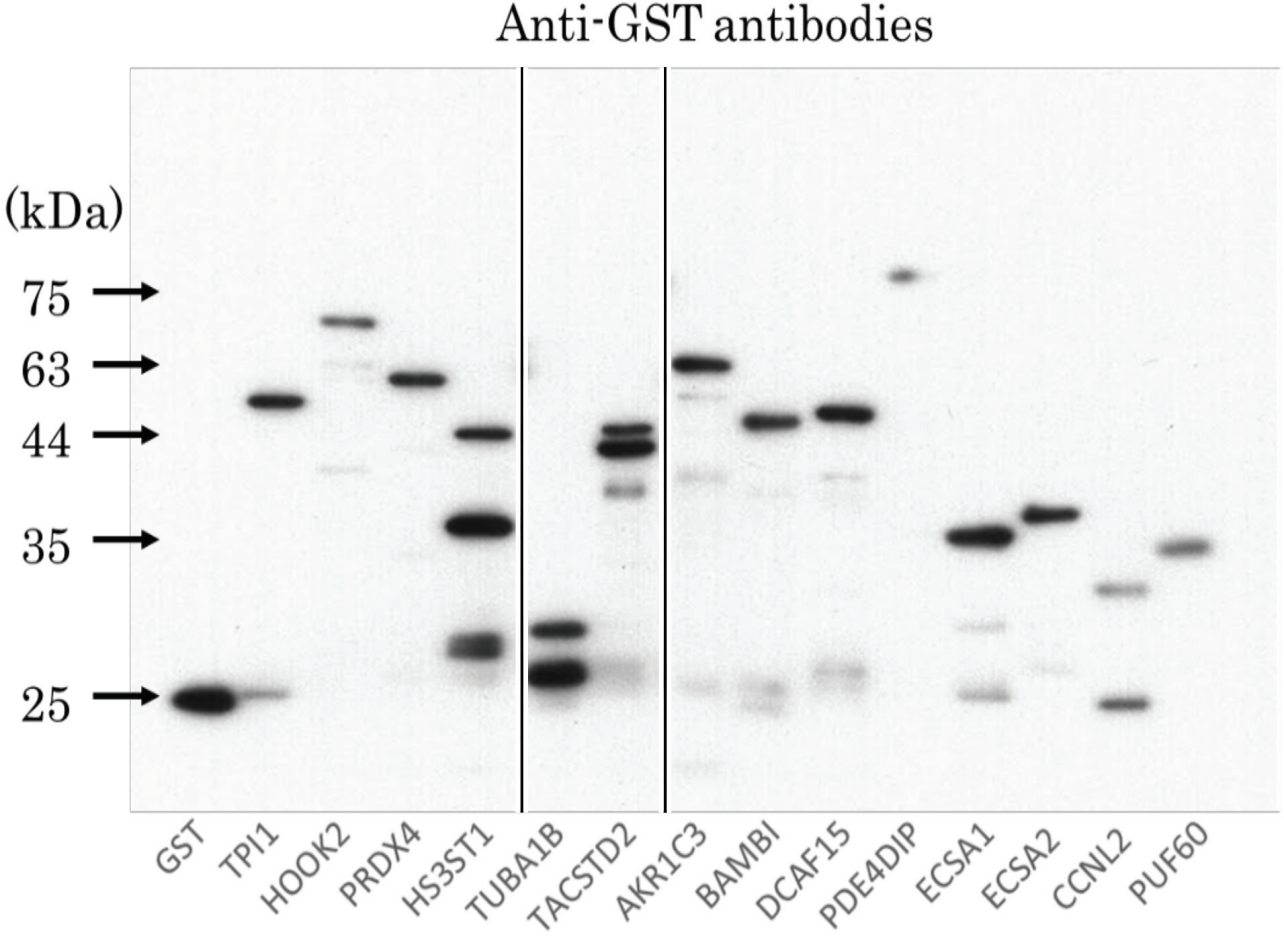
The presence of 14-antigens in patients with anti-GST antibodies To purify the SEREX-identified proteins, the insertion sequences of the 14 pBluescript plasmids were ligated in-frame into GST-tagged expression vectors. We confirmed by sequence analysis that the recombinant pGEX-4T-3 plasmids were properly recombined and GST-tagged recombinant proteins were affinity-purified using glutathione-Sepharose. To confirm the recombinant proteins to be the GST-tagged one that react with autologous plasma, the proteins were lysed in a SDS sample buffer, incubated at 100°C for 3 min, Affinity-purified GST-tagged antigens were separated on 11% SDS-polyacrylamide gels followed by Western blot using anti-GST antibody. All samples were examined simultaneously, at the same time on the same membrane.

The western blotting analysis confirmed the presence of auto-antibodies in the sera of patients. GST-TPI1, GST-HOOK2, GST-PRDX4, GST-HS3ST1, GST-TUBA1B, GST-TACSTD, GST-AKP1C3, GST-BAMBI, GST-DCAF1, GST-PDE4DIP, GST-ECSA1, GST-ECSA2, GST-CCNL2, and GST-PUF60 as well as GST proteins were recognized by anti-GST antibody as reactions of 27 kDa, 83 kDa, 60 kDa, 36 kDa, 29 kDa, 45 kDa, 63 kDa, 58 kDa, 60 kDa, 130 kDa, 36 kDa, 40 kDa, 25 kDa, 36 kDa, and 28-kDa proteins, respectively (Figure 1). On the contrary, only GST-ECSA1 or GST-ECSA2 was reacted with the serum antibodies of patients 14 and 17, respectively (Supplementary Figures 1A, 1B). Serum 19 showed the reactivity to TPI1 and HOOK2, which eventually enabled the recognition of both GST-TPI1 and GST-HOOK2 (Supplementary Figure 1C). Serum 20 showed the reactivity to AKP1C3, thereby it enabled the recognition of GST-AKP1C3 (Supplementary Figure 1D).

### Auto-antibody levels showed increase in patients with digestive organ cancers

**Figure 2:**
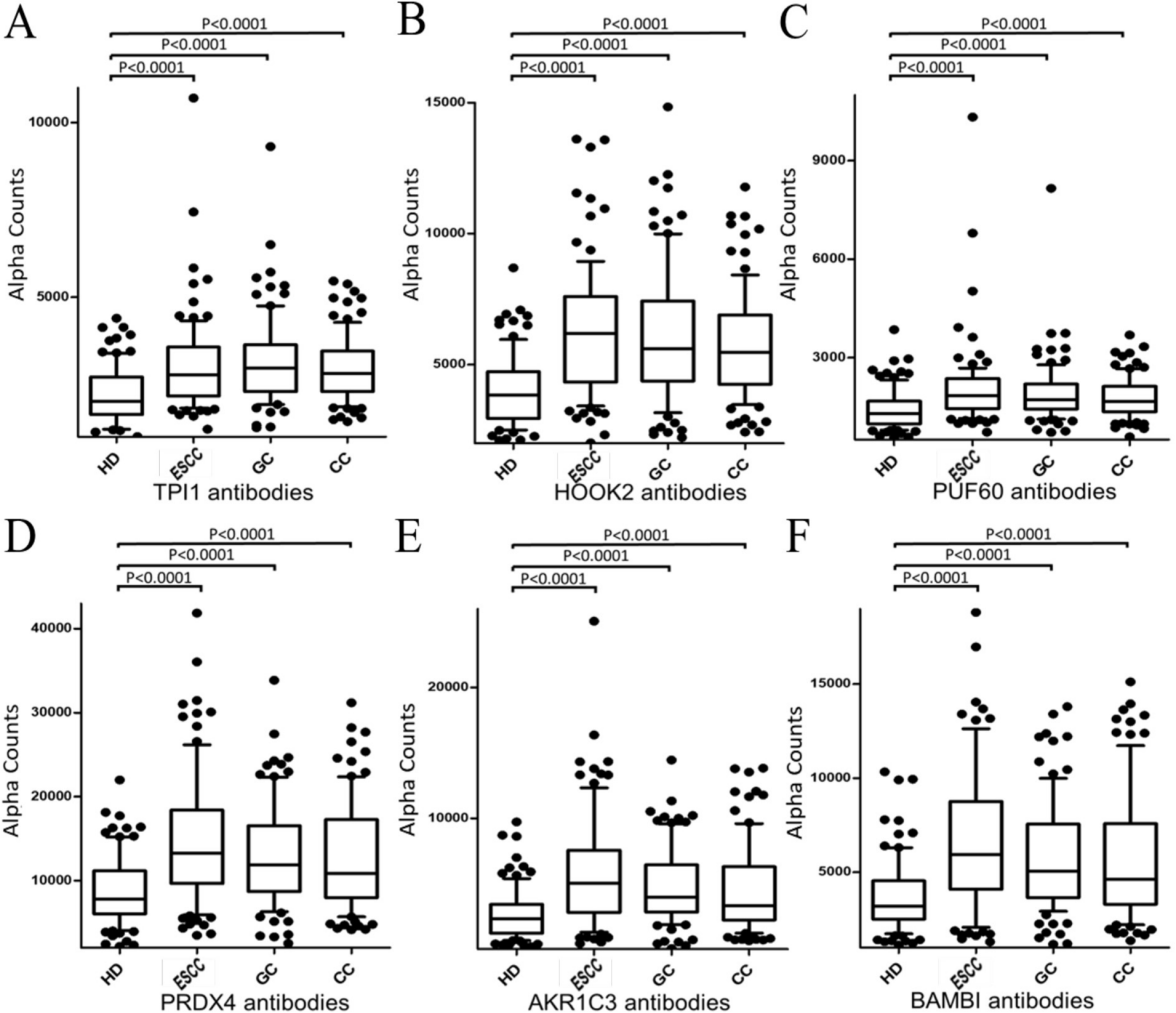
Comparison of levels of antibodies against top 6 SEREX antigens The levels of antibodies against TPI1, HOOK2, PUF60, PRDX4, AKR1C3, and BAMBI in healthy donors (HD), gastric cancer (GC), colon cancer (CC), and esophageal SCC (ESCC) examined by AlphaLISA are shown. Serum antibody levels examined by AlphalLISA are shown by a box-whisker plot. The box plots display the 10th, 20th, 50th, 80th and 90th percentiles. P values as compared to the HD specimens are shown. P values were calculated by Mann–Whitney U test.

**Figure 3:**
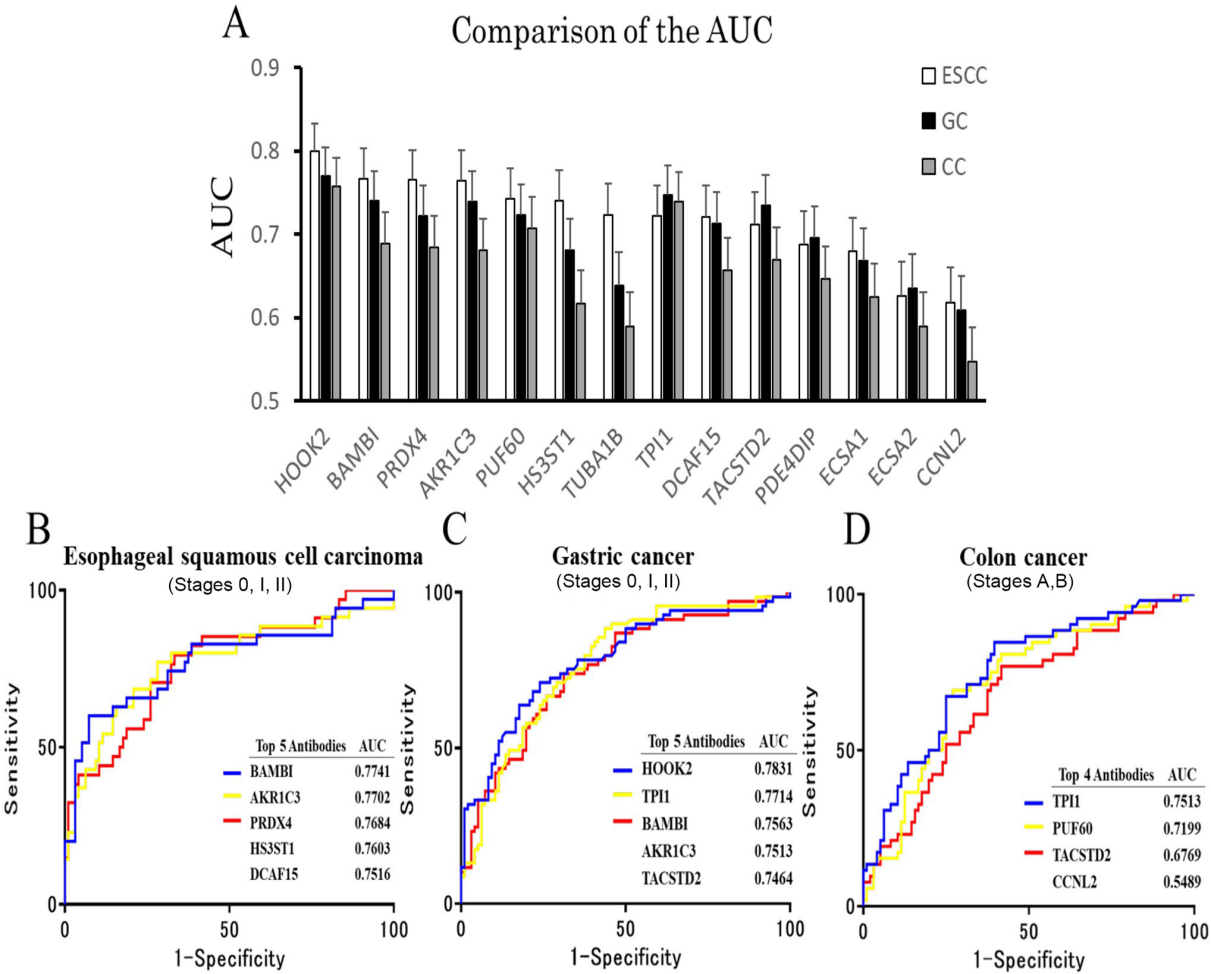
Comparison of the AUC by digestive organ cancer patients **(A)** The overall diagnostic efficiencies of 14 antibodies were evaluated by comparing the ROC curves. The area under each ROC analysis was calculated, and the difference between ROC curves was assessed the statistical significance. P < 0.05 was considered significant. ROC curves were generated, and the area under the curve (AUC) values were calculated using the GraphPad Prism 5. The error bars represent 95% CI (HD: n = 96, EC: n = 85, CC: n = 96, GC: n = 97). **(B, C, D)** The levels of ROC analysis in healthy donors (HD), gastric cancer (GC), colon cancer (CC), and esophageal SCC (ESCC) examined by AlphaLISA are shown. Candidate markers were shown higher top 3 AUC in early stage cancers than in progressive cancers.

We examined the levels of serum auto-antibodies using the sera of HD and patients with ESCC, GC, and CC procured from Chiba University Hospital. HD subjects from Higashi Funabashi Hospital were selected as healthy volunteers. The results of AlphaLISA showed that the levels of TPI1-Abs, HOOK2-Abs, PUF60-Abs, PRDX4-Abs, HS3ST1-Abs, TACSTD2-Abs, TUBA1B-Abs, AKR1C3-Abs, BAMBI-Abs, DCAF15-Abs, PDE4DIP-Abs, ECSA1-Abs, ECSA2-Abs, and CNNL2-Abs were significantly higher in patients with ESCC, GC, or CC than in HD (Figures 2A–2F and Supplementary Figures 2A–2H). The cutoff value was determined as the average + 2SD of HD, to keep the 95% confidence interval. Receiver operating curve (ROC) analysis was carried out to evaluate the ability of these markers to detect ESCC, GC, and CC. The areas under the curve (AUCs) of TPI1, HOOK2, PUF60, PRDX4, HS3ST1, TUBA1B, TACSTD2, AKR1C3, BAMBI, and DCAF15 for ESCC were significantly larger than 0.700 (Table 1). The highest AUC values were obtained for HOOK2 when compared with AUC values of ESCC. AUCs for GC were TPI1, HOOK2, PUF60, PRDX4, TACSTD2, AKR1C3, BAMBI and DCAF15 by larger than 0.700 respectively, whereas those for CC were TPI1, HOOK2, PUF60 by larger than 0.700 respectively (Figure 3A and Table 1). Next, gastrointestinal cancers were classified into early or advanced stages. Higher level of antibodies in early stages than in advanced stages cancers were shown. In AUC of ESCC showed BAMBI-0.7741, AKR1C3-0.7702, PRDX4-0.7684 (Figure 3B). In AUC of GC showed HOOK2-0.7831, TPl1-0.7714, BAMBI-0.7563 (Figure 3C). In AUC of CC showed TPl1-0.7513, PUF60-0.7199, TACSTD2-0.6769 (Figure 3D).

**Table 1:** Receiver operating curve (ROC) analysis of auto-antibodies examined in the sera of gastrointestinal cancers

	ESCC				GC				CC			
	Area	SE	95% CI	*P* value	Area	SE	95% CI	*P* value	Area	SE	95% CI	*P* value
HOOK2	0.8001	0.0324	0.7366 to 0.8637	< 0.0001	0.7695	0.0343	0.7022 to 0.8367	< 0.0001	0.7577	0.0343	0.6905 to 0.8249	< 0.0001
BAMBI	0.7664	0.0366	0.6947 to 0.8381	< 0.0001	0.7403	0.0358	0.6701 to 0.8105	< 0.0001	0.6883	0.0378	0.6142 to 0.7624	< 0.0001
PRDX4	0.7654	0.0353	0.6962 to 0.8347	< 0.0001	0.7221	0.0367	0.6502 to 0.7939	< 0.0001	0.6841	0.0380	0.6097 to 0.7585	< 0.0001
AKR1C3	0.7643	0.0363	0.6931 to 0.8355	< 0.0001	0.7393	0.0360	0.6688 to 0.8099	< 0.0001	0.6803	0.0381	0.6055 to 0.7550	< 0.0001
PUF60	0.7428	0.0368	0.6706 to 0.8151	< 0.0001	0.7231	0.0370	0.6507 to 0.7956	< 0.0001	0.7075	0.0377	0.6336 to 0.7815	< 0.0001
HS3ST1	0.7398	0.0374	0.6665 to 0.8130	< 0.0001	0.6805	0.0383	0.6054 to 0.7556	< 0.0001	0.6165	0.0404	0.5372 to 0.6957	0.0052
TUBA1B	0.7227	0.0378	0.6486 to 0.7969	< 0.0001	0.6384	0.0398	0.5603 to 0.7165	0.0009	0.5891	0.0408	0.5090 to 0.6691	0.0325
TPI1	0.7218	0.0373	0.6486 to 0.7949	< 0.0001	0.7470	0.0354	0.6777 to 0.8163	< 0.0001	0.7389	0.0356	0.6692 to 0.8086	< 0.0001
DCAF15	0.7209	0.0381	0.6462 to 0.7955	< 0.0001	0.7131	0.0370	0.6405 to 0.7856	< 0.0001	0.6564	0.0391	0.5798 to 0.7329	0.0002
TACSTD2	0.7119	0.0385	0.6363 to 0.7874	< 0.0001	0.7348	0.0361	0.6640 to 0.8056	< 0.0001	0.6691	0.0391	0.5925 to 0.7457	< 0.0001
PDE4DIP	0.6875	0.0398	0.6095 to 0.7655	< 0.0001	0.6960	0.0377	0.6220 to 0.7700	< 0.0001	0.6459	0.0397	0.5680 to 0.7238	0.0005
ECSA1	0.6797	0.0396	0.6021 to 0.7572	< 0.0001	0.6687	0.0389	0.5926 to 0.7449	< 0.0001	0.6247	0.0401	0.5461 to 0.7033	0.0028
ECSA2	0.6257	0.0417	0.5439 to 0.7075	0.0035	0.6354	0.0404	0.5561 to 0.7147	0.0012	0.5889	0.0414	0.5077 to 0.6701	0.0329
CCNL2	0.6179	0.0420	0.5355 to 0.7003	0.0062	0.6092	0.0410	0.5289 to 0.6896	0.0090	0.5464	0.0418	0.4644 to 0.6284	0.2655

SE: Standard error.

95% CI: Adjusted 95% confidence interval.

P value: Mann-Whitney U test.

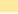
>0.700 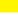
>0.750.

### Combined ROC curve increases AUC

**Figure 4:**
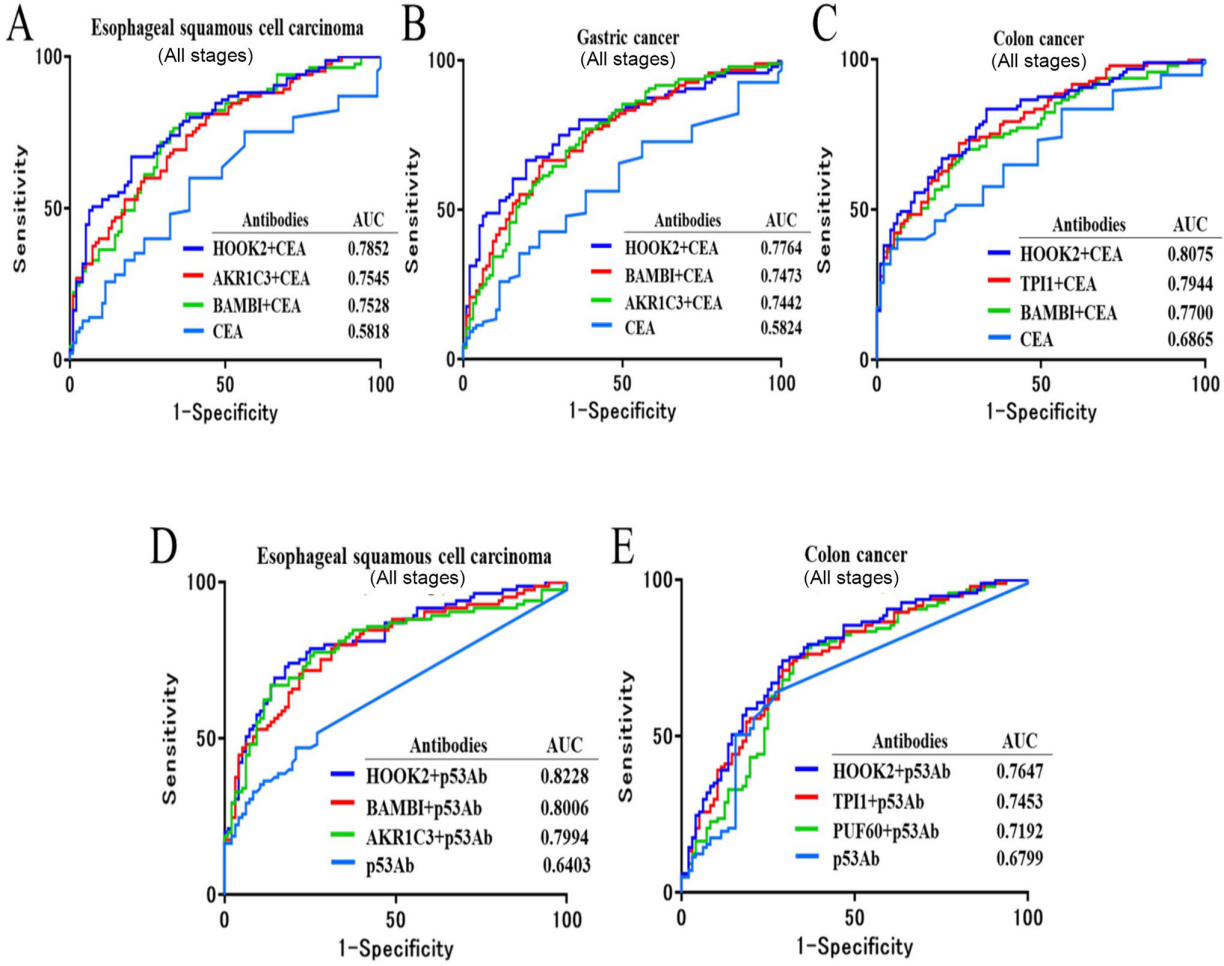
ROC analysis in combination of candidate markers and CEA/p53 antibody markers The ROC analysis of detected candidate markers and two clinically used tumor markers (anti-p53 antibody, CEA) were created on the basis of the Z scores data normalized with standard deviation to the quantified Alpha count data of 277 patients with various cancers and 96 healthy subjects. Shown in descending order of AUC. Target number: HD = 96, EC = 85, CC = 97, GC = 96. Show the top three antibody lists.

The receiver operating combined curve (ROC) analysis was carried out to evaluate the facility of these markers to detect ESCC, GC, and CC (Figure 4 and Supplementary Figure 3). The antibodies with CEA, only the AUC of DCAF15+CEA increased to 0.7223 in ESCC (Figure 4A and Supplementary Figure 3A). However, the AUC of TPI1+CEA decreased to 0.7435 in GC (Figure 4B and Supplementary Figure 3B). Further, in CC, HOOK2+CEA showed AUC of 0.8075 (Figure 4C and Supplementary Figure 3C). The AUC of all the antibodies combined with anti-p53 antibody, HOOK2 and BAMBI showed 0.8228 in ESCC (Figure 4D and Supplementary Figure 3D). There was no antibody indicating AUC larger than 0.800 in CC (Figure 4E and Supplementary Figure 3E). The AUC was summarized in early or advanced stages cancers were showed in Table 2. Advanced stage cancers showed higher AUC than that of early stage cancers. Further, in ESCC and CC, the efficiency of early diagnosis was increased when combined with HOOK2 and anti-p53 antibody (AUC of ESCC: 0.7985, AUC of CC: 0.7669).

**Table 2:** The largest AUC of single or combined auto-antibodies in gastrointestinal cancers depending on the early or advanced stages

	Early stages (0, I, II or A, B)		Advanced stages (III, IV or C, D)	
	Single AUC	Combined AUC	Single AUC	Combined AUC
ESCC	HOOK2: 0.7804	CEA+BAMBI: 0.7631	HOOK2: 0.8119	CEA+HOOK2: 0.7844
		p53 Ab+HOOK2: 0.7985		p53 Ab+HOOK2: 0.8365
GC	HOOK2: 0.7831	CEA+HOOK2: 0.7844	ECSA1: 0.7446	CEA+ECSA1: 0.7904
CC	HOOK2: 0.7536	CEA+HOOK2: 0.7406	HOOK2: 0.7670	CEA+HOOK2: 0.9023
		p53 Ab+HOOK2: 0.7669		p53 Ab+HOOK2: 0.7889

## DISCUSSION

We have identified novel potential diagnostic markers for digestive organ cancers by SEREX screening. Those serum antibody markers were detected using purified GST-fusion proteins as antigen. Two hundred seventy-seven patients with various cancers were evaluated for the presence of various-Abs. Patients with confirmed digestive organ cancers demonstrated significantly higher levels of the antibodies against most ESCC SEREX antigens. This suggested that ESCC, GC, and CC have a common carcinogenesis process. However, some of the antibody markers showed differential antibody levels, i.e., all 14 markers were significantly higher in patients with ESCC or GC in comparison to those in HDs. On the contrary, the levels of ECSA2-Ab and CCNL2-Ab were not significantly different between patients with CC and HDs (Figure 2 and Supplementary Figure 2). Similar results were also attained by ROC analysis. The AUCs were higher than 0.6 for most of the markers except ECSA2 and CCNL2 in comparison to CC (Table 1). Also, the AUCs higher than 0.7 were observed for ten markers versus ESCC, eight markers versus GC, and three markers versus CC. Thus, tumor nature of ESCC might be more similar to GC than to CC. It is conceivable that HOOK2-Ab, PUF60-Ab, and TPI1-Ab are common markers for digestive organ cancers.

Furthermore, the combined ROC analysis of antibodies with anti-p53 antibody, and CEA showed elevation in AUCs of almost antibodies in various cancers. The combinations of anti-p53 antibody and HOOK2 markers were valuable for early detection of ESCC and CC (Figure 4 and Table 2). Therefore, the combination of antibodies with anti-p53 antibody and CEA is a potential approach for the diagnosis of digestive organ cancers. However, prospective multi-institutional studies comparing the sensitivity and specificity of this combinational detection approach are necessary.

AlphaLISA is an excellent method for measuring antibody levels as compared to ELISA because it has low variations, stable background, and high specificity. It does not involve plate-washing steps, but instead involves mixing of antigens with antibodies in sera followed by the addition of donor and acceptor beads. For instance, Figure 2 showed highly reproducible results, including distributions, P values, and positive rates despite using different sets of sera from healthy donors and patients. The precise measurement offered by AlphaLISA might enable establishment of antibody markers, although most of the existing tumor diagnosis methods involved antigen markers, with the exception of the anti-p53 marker [2, 15]. The measurement of antibodies was more sensitive in comparison to the measurement of the antigen levels owing to the stability of IgG proteins and their amplification by repeated exposures to antigenic proteins. Prior to development, highly-malignant tumors could induce necrosis, leading to exposure of intracellular antigenic proteins to plasma. Therefore, using combinational antibody detection approach might enable precise tumor diagnosis. In this study, we examined and proposed some of these candidate markers for the early diagnosis of ESCC, CC, and GC. As per our knowledge, no other studies have suggested such kind of candidate markers in digestive organ cancers. Our study would be an important approach for further selecting diagnostic marker candidates for digestive organ cancers.

## MATERIALS AND METHODS

### Clinical samples

The present study was performed in accordance with “The Code of Ethics of the World Medical Association” (Declaration of Helsinki). The Local Ethical Review Board of the Chiba University, Graduate School of Medicine, and those of co-operating hospitals approved this work. Sera of patients with ESCC (n = 85), GC (n = 96), and CC (n = 97) were obtained from the Department of Frontier Surgery, Chiba University Hospital, Chiba, Japan (Supplementary Tables 1–5). Sera of health donors (HDs) (n = 96) were obtained from Higashi Funabashi Hospital. Written informed consent was obtained from all participants prior to this study. Each serum sample was centrifuged at 2,000 × g for 10 min and then the supernatant was stored at −80°C until use. Repeated thawing and freezing of samples were avoided.

### Screening by expression cloning

Recombinant DNA studies were performed with the official permission of the Chiba University Graduate School of Medicine and were carried out in accordance with the rules of the Japanese government. We used a λZAP II phage cDNA library prepared from the mRNA of the T.Tn cells and a commercially available human fetal testis cDNA library (Uni-ZAP XR Premade Library, Stratagene, La Jolla, CA) to screen for clones that were immunoreactive against serum IgG from patients with ESCC as described in earlier studies [16, 17]. *Escherichia coli* XL1-Blue MRF' was infected with λZAP II or Uni-ZAP XR phage and the expression of resident cDNA clones was induced after blotting the infected bacteria onto NitroBind nitrocellulose membranes (Osmonics, Minnetonka, MN) The above membranes had been treated with 10 mM isopropyl-β-D-thiogalactoside (IPTG, Wako Pure Chemicals, Osaka, Japan) for 30 min. The membranes with bacterial proteins were rinsed 3 times with TBS-T [20 mM Tris–HCl (pH 7.5), 0.15 M NaCl, and 0.05% Tween-20], and non-specific binding was blocked by incubation with 1% protease-free bovine serum albumin (Nacalai Tesque, Inc., Kyoto, Japan) in TBS-T for 1 h. The membranes were exposed to 1:2000-diluted sera of patients for 1 h. After three washes with TBS-T, the membranes were incubated for 1 h with 1:5000-diluted alkaline phosphatase-conjugated goat anti-human IgG (Jackson ImmunResearch Laboratories, West Grove, PA). Positive reactions were developed using 100 mM Tris–HCl (pH 9.5) containing 100 mM NaCl, 5 mM MgCl_2_, 0.15 mg/mL of 5-bromo-4-chloro-3-indolylphosphate, and 0.3 mg/mL of nitro blue tetrazolium (Wako Pure Chemicals). Positive clones were re-cloned twice until obtaining monoclonality as described in previous studies [16, 18, 19].

Monoclonal phage cDNA clones were converted to pBluescript phagemids by excision *in vivo* using the ExAssist helper phage (Stratagene). Plasmid pBluescript containing cDNA was obtained from the *E. coli* SOLR strain after transformation by the phagemid. The sequences of cDNA inserts were evaluated for homology with identified genes or proteins within the public sequence database (http://blast.ncbi.nlm.nih.gov/Blast.cgi).

### Expression and purification of antigen proteins

The expression plasmids of glutathione S- transferase (GST)-fused proteins were constructed by recombining the cDNA sequences into pGEX-4T-3 (GE Healthcare Life Sciences, Pittsburgh, PA). The inserted DNA fragments were ligated in frame to pGEX-4T-3 using the Ligation Convenience Kits (Nippon Gene, Toyama, Japan). Ligation mixtures were used to transform ECOS™-competent *E. coli* BL-21 (Nippon Gene), and appropriate recombinants were confirmed by DNA sequencing as well as protein expressions. Treating the transformed *E. coli* with 0.1 mM IPTG for 3 h induced the expression of the GST-fusion proteins. The GST-fused recombinant proteins were purified by Glutathione Sepharose column chromatography according to the manufacturer's instructions (GE Healthcare Life Sciences) and dialyzed against phosphate-buffered saline as described in previous studies [20–22].

### Western blotting

GST, GST-TPI1, GST-HOOK2, GST-PRDX4, GST-HS3ST1, GST-TUBA1B, GST-TACSTD2, GST-AKP1C3, GST-BAMBI, GST-DCAF1, GST-PDE4DIP, GST-ECSA1, GST-ECSA2, GST-CCNL2, and GST-PUF60 proteins (0.3 μg) were electrophoresed through SDS-polyacrylamide gel followed by western blotting using anti-GST (Rockland, Gilbertsville, PA) or sera from patients with ESCC (No.14, No.17, No.19, and No.20). After incubation with horseradish peroxidase-conjugated secondary antibody, immunoreactivity was detected with the Immobilon (Merck Millipore, Darmstadt, Germany) as described in previous studies [23, 24].

### AlphaLISA (amplified luminescence proximity homogeneous assay)

AlphaLISA was performed using 384-well microtiter plates (white opaque OptiPlate™, Perkin Elmer) containing 2.5 μL of 1/100-diluted sera and 2.5 μL of GST or GST-fusion proteins (10 μg/mL) in AlphaLISA buffer (25 mM HEPES, pH 7.4, 0.1% casein, 0.5% Triton X-100, 1 mg/mL dextran-500, and 0.05% Proclin-300). The reaction mixture was incubated at room temperature for 6–8 h. Next, anti-human IgG-conjugated acceptor beads (2.5 μL of 40 μg/mL) and glutathione-conjugated donor beads (2.5 μL of 40 μg/mL) were added and incubated further for 7–21 days at room temperature in the dark. The chemical emission was read on an EnSpire Alpha microplate reader (PerkinElmer) as previously described [25–31]. Specific reactions were calculated by subtracting Alpha values of GST control from the values of GST-fusion proteins. AlphaLISA is a registered trademark of PerkinElmer, Inc., which is shown in a website ‘ http://www.perkinelmer.com/lab-solutions/resources/docs/GDE_ELISA-to-AlphaLISA.pdf’.

### Statistical analyses

All statistical analyses were carried out using the GraphPad Prism 5 (GraphPad Software, La Jolla, CA). Mann–Whitney U test was used to determine the significance of the differences between the two groups. The predictive values of markers for diseases were assessed by receiver operating curve (ROC) analysis and the cutoff values were set at the values that maximize the sums of the sensitivity and specificity. All tests were two-tailed and a *P* value below 0.05 was considered significant. We calculated antibody group-specific Z-scores for these measures to facilitate the comparison across anti-p53 antibody, CEA and antibodies groups. Z-score analysis was performed after normalization to healthy donors mean values:

Z-score = [(control mean) - (individual value)] / (control SD) [32, 33].

The Combined ROC analysis was performed by adding each Z score.

## SUPPLEMENTARY MATERIALS FIGURES AND TABLES









## Abbreviations

AUC: area under the curve
GST: glutathione S- transferase
HD: healthy donor
IPTG: isopropyl-β-D-thiogalactoside
ROC: receiver operating curve
SEREX: serological identification of antigens by recombinant cDNA expression cloning
