# Supplementary material for: Identification of specific and common diagnostic antibody markers for gastrointestinal cancers by SEREX screening using testis cDNA phage library
Source: Oncotarget. 2018 Jan 1;9(26):18559–69. doi: 10.18632/oncotarget.24963 (PMC5915093; doi:10.18632/oncotarget.24963)
Supplement: Supplementary file 1 [file oncotarget-09-18559-s001.pdf]

Identification of specific and common diagnostic antibody markers for gastrointestinal cancers by SEREX screening using testis cDNA phage library

SUPPLEMENTARY MATERIALS

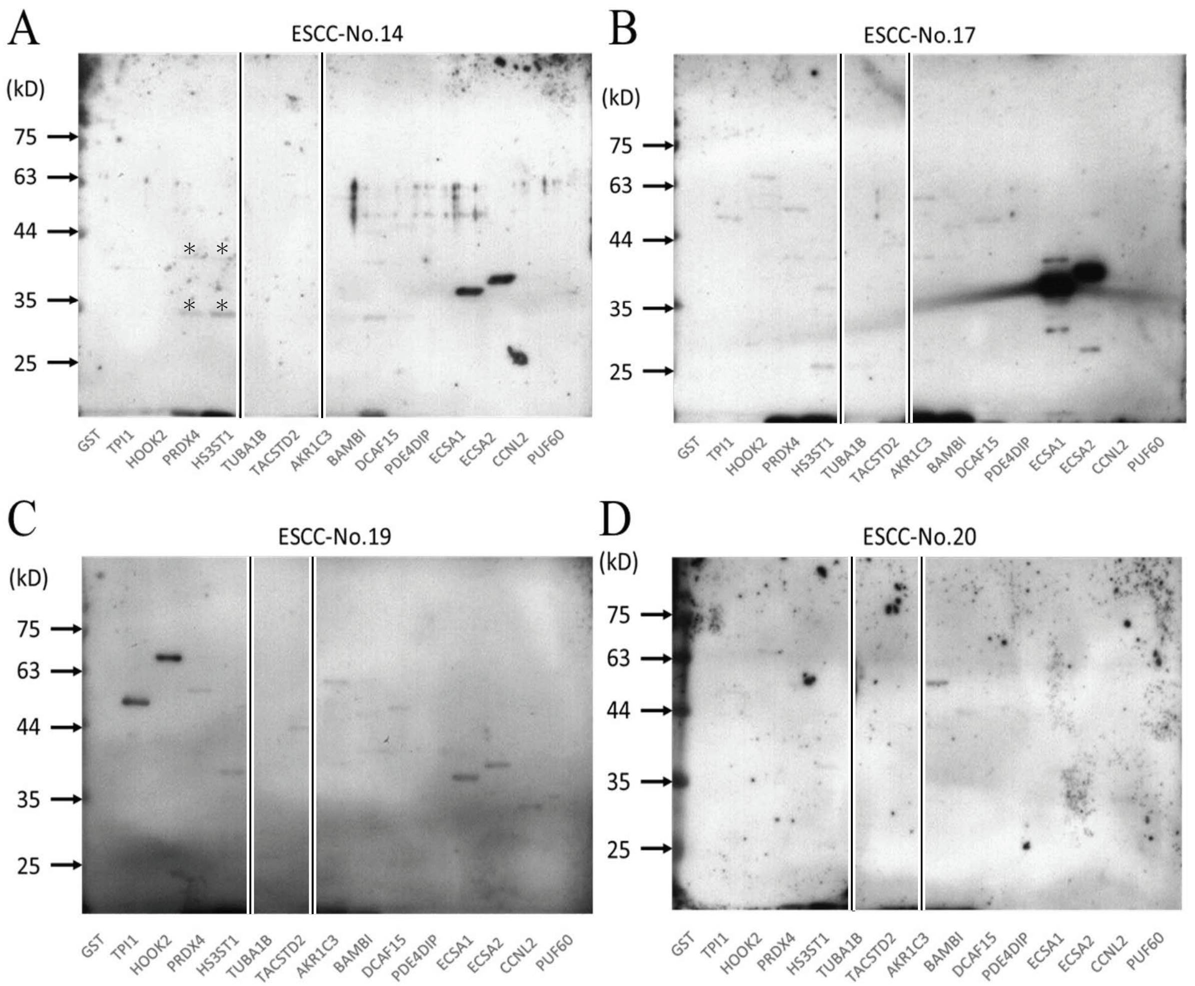

Supplementary Figure 1: The presence of 14 antibodies in patients with esophageal SCC. To purify the SEREX-identified proteins, the insertion sequences of the 14 pBluescript plasmids were ligated in-frame into GST-tagged expression vectors. We confirmed by sequence analysis that the recombinant pGEX-4T-3 plasmids were properly recombined and GST-tagged recombinant proteins were affinity-purified using glutathione-Sepharose. To confirm the recombinant proteins to be the GST-tagged one that react with autologous plasma, the proteins were lysed in a SDS sample buffer, incubated at 100°C for 3 min, GST and GST-fusion proteins (0.3 µg) were electrophoresed through 11% SDS-polyacrylamide gel electrophoresis followed by western blot analysis using sera of esophageal cancer patient EC-No.14, EC-No.17, EC-No.19, and EC-No.20. *: nonspecific reaction.

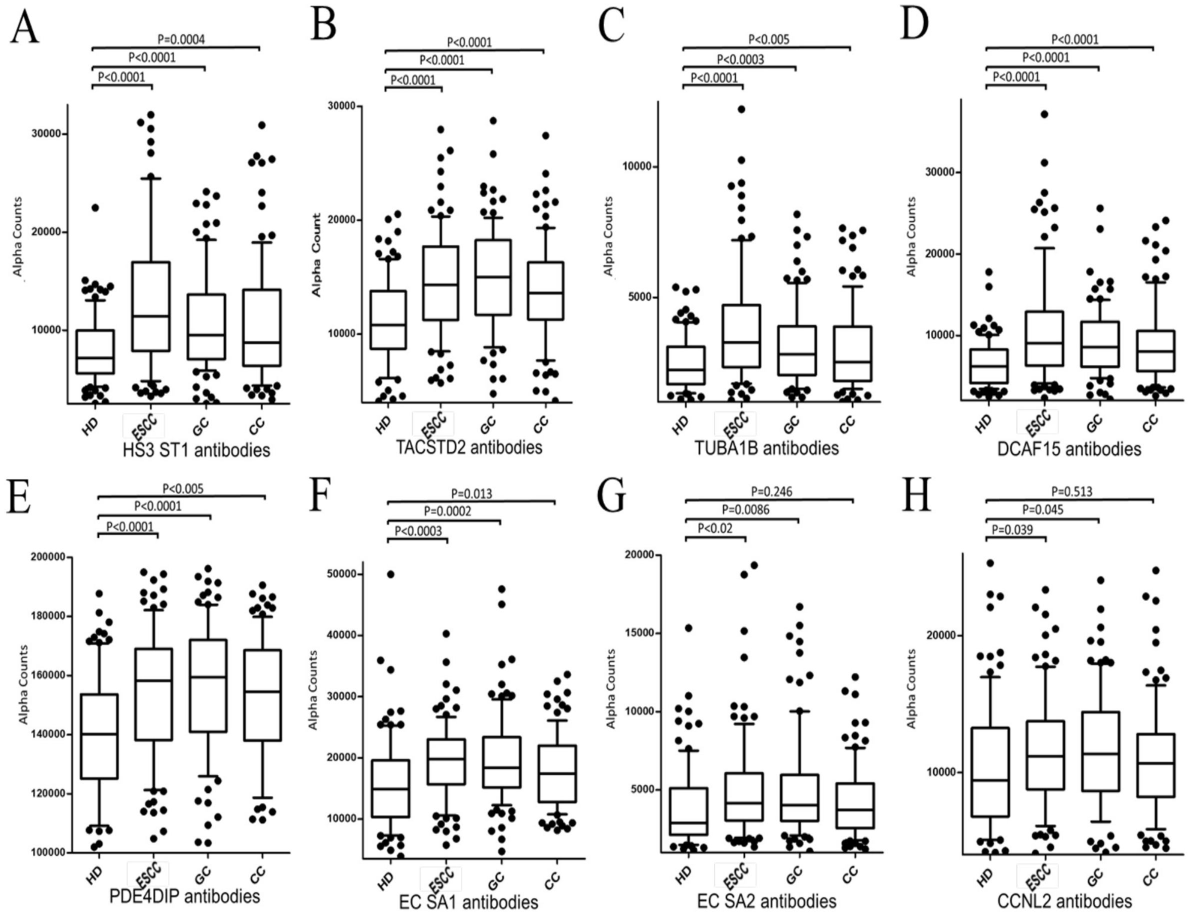

Supplementary Figure 2: Comparison of levels of antibodies against 8 SEREX antigens. The levels of antibodies against HS3ST1, TACSTD2, TUBA1B, DCAF15, PDE4DIP, ECSA1, ECSA2, and CCNL2 in healthy donors (HD), gastric cancer (GC), colon cancer (CC), and esophageal SCC (EC) examined by AlphaLISA are shown. Serum antibody levels examined by AlphalLISA are shown by a box-whisker plot. The box plots display the 10th, 20th, 50th, 80th and 90th percentiles. P values as compared to the HD specimens are shown. P values were calculated by Mann–Whitney U test.

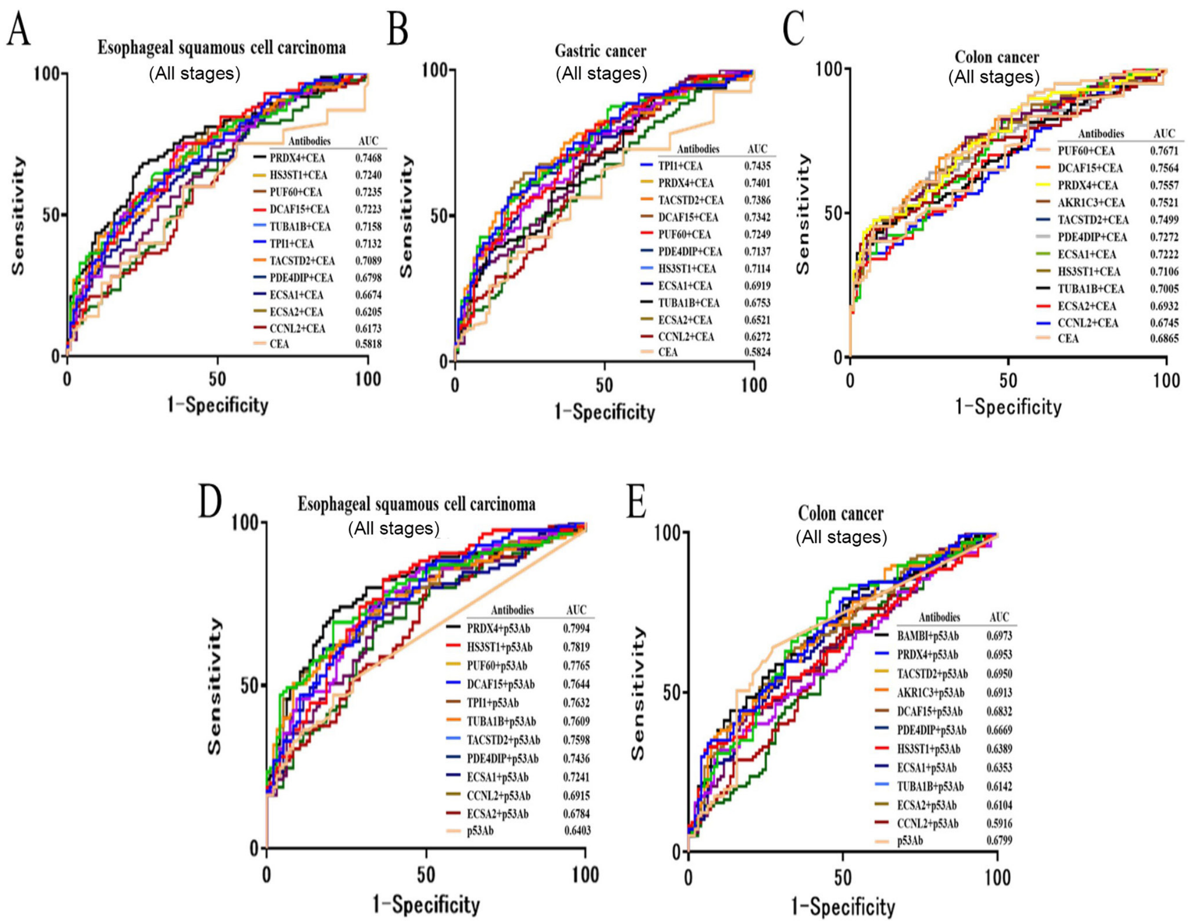

Supplementary Figure 3: ROC analysis in combination of candidate markers and CEA/p53 antibody markers. The ROC analysis of detected candidate markers and two clinically used tumor markers (anti-p53 antibody, CEA) were created on the basis of the Z scores data normalized with standard deviation to the quantified Alpha count data of 277 patients with various cancers and 96 healthy subjects. Shown in descending order of AUC. Target number: HD = 96, EC = 85, CC = 97, GC = 96. Show the 11 antibody lists.

Supplementary Table 1: Definition of Stages of ESCC patients in this study according to Japanese Classification of Esophageal Cancer

Stage

<table>
<tr><th></th><th>N0</th><th>N1</th></tr>
<tr><td>Tis</td><td>0</td><td></td></tr>
<tr><td>T1</td><td>I</td><td rowspan="2">IIB</td></tr>
<tr><td>T2</td><td rowspan="2">IIA</td></tr>
<tr><td>T3</td><td rowspan="2">III</td></tr>
<tr><td>T4</td><td>III</td></tr>
<tr><td>M1a</td><td colspan="2">IVA</td></tr>
<tr><td>M1b</td><td colspan="2">IVB</td></tr>
</table>

Tis: intraepithelial cancer.
T1: lamina propria mucosae or submucosal invasion.
T2: muscularis propria invasion.
T3: adventitial invasion.
T4: surrounding tissue invasion.
N0: no lymph node metastasis.
N1: lymph node metastasis.
M1a: metastasis within neck lymph nodes.
M1b: metastasis to other organs.

Supplementary Table 2: Definition of Stages of GC patients in this study according to Japanese Classification of Gastric Cancer

Stage

	N0	N1	N2	N3
Tis	**0**			
T1	**Ia**	**Ib**	**II**	**IV**
T2a	**Ib**	**II**	**IIIa**	**IV**
T2b	**Ib**	**II**	**IIIa**	**IV**
T3	**II**	**IIIa**	**IIIb**	**IV**
T4	**IIIa**	**IV**	**IV**	**IV**
M1	**IV**	**IV**	**IV**	**IV**

Tis: intraepithelial cancer.
T1: lamina propria mucosae or submucosal invasion.
T2: muscularis propria invasion or submucosal invasion.
T2a: muscularis propria invasion.
T2b: submucosal invasion.
T3: adventitial invasion.
T4: Tumour invades adjacent structures.
N0: no lymph node metastasis.
N1: 1-6 metastatic lymph nodes.
N2: 7-15 metastatic lymph nodes.
N3: >16 metastatic lymph nodes.
M1: metastasis.

Supplementary Table 3: The clinical features of esophageal squamous cell carcinoma patients

See Supplementary File 1

Supplementary Table 4: The clinical features of colon cancer patients

See Supplementary File 2

Supplementary Table 5: The clinical features of gastric cancer patients

See Supplementary File 3
